# Presaccadic Attention Enhances and Reshapes the Contrast Sensitivity Function Differentially around the Visual Field

**DOI:** 10.1523/ENEURO.0243-24.2024

**Published:** 2024-09-10

**Authors:** Yuna Kwak, Yukai Zhao, Zhong-Lin Lu, Nina Maria Hanning, Marisa Carrasco

**Affiliations:** ^1^Department of Psychology, New York University, New York, New York 10003; ^2^Center for Neural Science, New York University, New York, New York 10003; ^3^Division of Arts and Sciences, New York University Shanghai, Shanghai 200124, China; ^4^NYU-ECNU Institute of Brain and Cognitive Science, Shanghai 200062, China; ^5^Department of Psychology, Humboldt-Universität zu Berlin, Berlin 12489, Germany

**Keywords:** contrast sensitivity, contrast sensitivity function, perceptual asymmetries, polar angle, presaccadic attention, saccade

## Abstract

Contrast sensitivity (CS), which constrains human vision, decreases from fovea to periphery, from the horizontal to the vertical meridian, and from the lower vertical to the upper vertical meridian. It also depends on spatial frequency (SF), and the contrast sensitivity function (CSF) depicts this relation. To compensate for these visual constraints, we constantly make saccades and foveate on relevant objects in the scene. Already before saccade onset, presaccadic attention shifts to the saccade target and enhances perception. However, it is unknown whether and how it modulates the interplay between CS and SF, and if this effect varies around polar angle meridians. CS enhancement may result from a horizontal or vertical shift of the CSF, increase in bandwidth, or any combination. In addition, presaccadic attention could enhance CS similarly around the visual field, or it could benefit perception more at locations with poorer performance (i.e., vertical meridian). Here, we investigated these possibilities by extracting key attributes of the CSF of human observers. The results reveal that presaccadic attention (1) increases CS across SF, (2) increases the most preferred and the highest discernable SF, and (3) narrows the bandwidth. Therefore, presaccadic attention helps bridge the gap between presaccadic and postsaccadic input by increasing visibility at the saccade target. Counterintuitively, this CS enhancement was more pronounced where perception is better—along the horizontal than the vertical meridian—exacerbating polar angle asymmetries. Our results call for an investigation of the differential neural modulations underlying presaccadic perceptual changes for different saccade directions.

## Significance Statement

The contrast sensitivity function (CSF) describes how perception of contrast depends on spatial frequency. Contrast sensitivity is highest at the fovea and decreases in the periphery, especially at locations along the vertical meridian. We thus make saccadic eye movements to view objects in detail. Already before eye movements, presaccadic attention enhances perception at the target location. But how does it influence the interplay between contrast sensitivity and spatial frequency, and does its effect vary around the visual field? Using hierarchical Bayesian modeling, we show that presaccadic attention enhances and reshapes the CSF to prepare the periphery for upcoming fixation. Interestingly, it does so more at horizontal locations where vision is stronger, suggesting smoother perception across horizontal than vertical eye movements.

## Introduction

**Figure 1.**
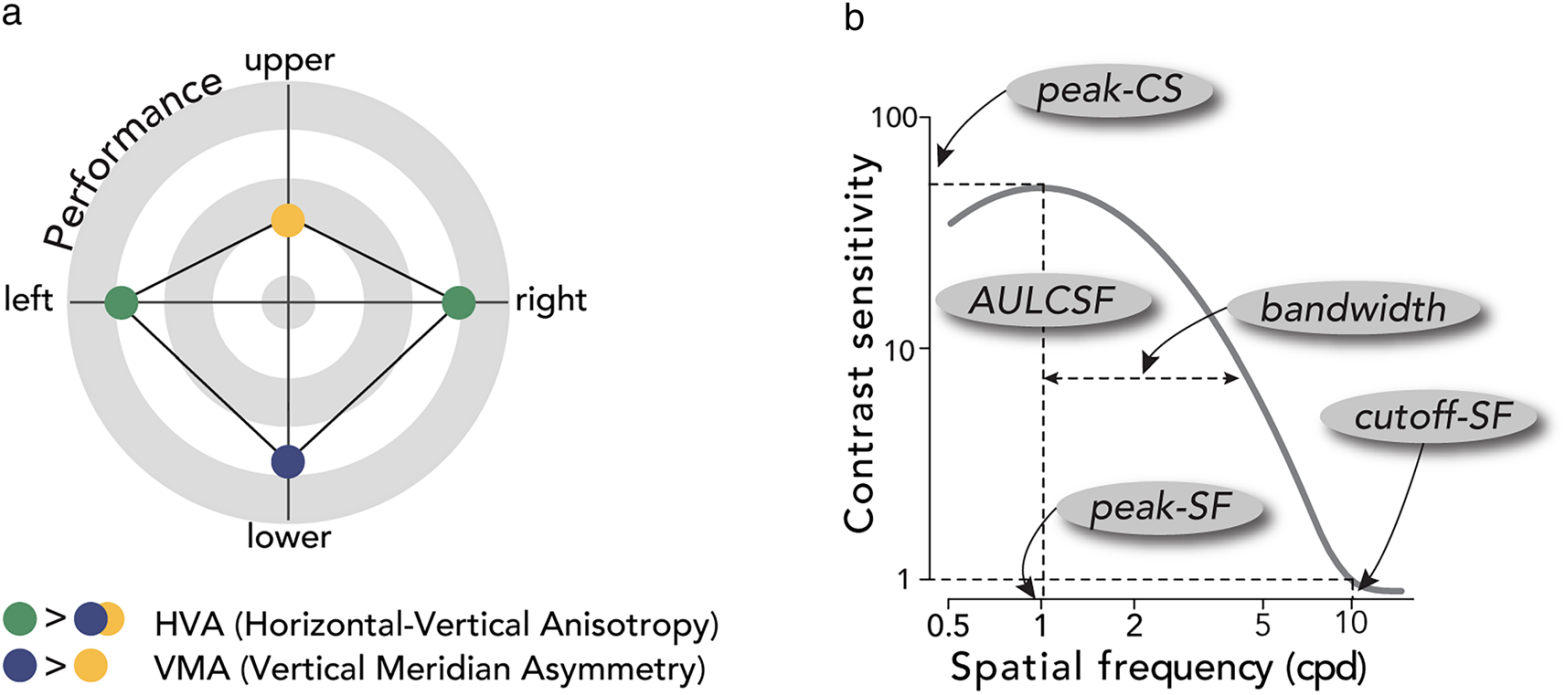
Background. ***a***, Polar angle asymmetries around the visual field. Visual performance is better along the horizontal than the vertical meridian, and along the lower than the upper vertical meridian. ***b***, Key attributes of the CSF. We derived peak-CS (peak contrast sensitivity), peak-SF (peak spatial frequency), bandwidth (full width at half maximum; half width shown for illustration purposes), cutoff-SF (cutoff spatial frequency), and AULCSF (area under the log CSF). Key CSF attributes were measured during fixation and saccade preparation to quantify presaccadic attention modulation on the CSFs.

Visual processing varies across eccentricity and around polar angle locations. Our ability to process fine details at the center of gaze declines with eccentricity (Frisén and Glansholm, 1975). Moreover, at iso-eccentric locations, visual performance changes as a function of polar angle (Fig. 1*a*, review: Himmelberg et al., 2023): It is better along the horizontal than vertical meridian (HVA, horizontal–vertical anisotropy) and along the lower vertical than the upper vertical meridian (VMA, vertical meridian asymmetry).

We often compensate for these visual constraints by making rapid saccadic eye movements to actively explore the world and place relevant objects in the fovea where visual perception is most sensitive (Eckstein, 2017). Already before our eyes move (during saccade preparation), presaccadic attention shifts to the saccade target location and prioritizes visual processing (Kowler et al., 1995; Deubel and Schneider, 1996; Montagnini and Castet, 2007). Presaccadic attention boosts accuracy (Hoffman and Subramaniam, 1995; Hanning et al., 2019) and reduces reaction time (Shepherd et al., 1986). Some studies investigating how presaccadic attention modulates perception of basic visual dimensions have revealed that saccade preparation enhances contrast sensitivity (Li et al., 2021b; Hanning et al., 2022, 2023, 2024) and spatial acuity (Kwak et al., 2023); sharpens orientation tuning and orientation acuity (Castet et al., 2006; Li et al., 2016; Ohl et al., 2017); and shifts spatial frequency (SF) tuning by enhancing sensitivity to higher SFs (Li et al., 2016, 2019). The presaccadic attention effect increases as saccade onset approaches (Rolfs and Carrasco, 2012; Li et al., 2016; Ohl et al., 2017; Hanning et al., 2019; Kroell and Rolfs, 2021). These studies suggest a functional role of presaccadic attention in rendering visual information at the peripheral saccade target more “fovea-like” than it was before saccade preparation. Across every saccade, two distinct percepts need to be seamlessly integrated: a blurry presaccadic input from the peripheral saccade target and a high-resolution postsaccadic input from the same location once the saccade target has been foveated. Presaccadic attention narrows the difference between and eases the integration of pre- and postsaccadic visual information, facilitating perceptual continuity (Herwig, 2015; Stewart et al., 2020; Li et al., 2021a).

Contrast sensitivity constrains our vision. It drops markedly with eccentricity (Rovamo et al., 1978; Virsu and Rovamo, 1979) and exhibits pronounced polar angle asymmetries (Rijsdijk et al., 1980; Abrams et al., 2012; Baldwin et al., 2012; Jigo et al., 2023). Moreover, contrast sensitivity depends heavily on SF and is bandpass (Fig. 1*b*). At the fovea, for example, it peaks at a SF range of 2–6 cpd and declines rapidly for lower and higher SFs (Campbell and Robson, 1968; Kelly, 1977). In addition to eccentricity and polar angle, the CSF varies with stimulus size (Rovamo et al., 1993), background luminance (Rovamo et al., 1978), and temporal frequency (Robson, 1966). This general relation between contrast sensitivity and SF, referred to as the contrast sensitivity function (CSF), defines our “window of visibility” (Watson et al., 1986).

Presaccadic attention has been shown to increase contrast sensitivity across the contrast response function at a constant SF (M. Zhao et al., 2012; Li et al., 2021b; Hanning et al., 2022), and performance at a range of SFs at a constant contrast (Kroell and Rolfs, 2021). But how it modulates the interplay between contrast and SF sensitivity is unknown: An increase in contrast sensitivity at a given SF may result from an upward shift of the CSF, a horizontal shift, increased bandwidth, or any of their combinations (Fig. 1*b*). Furthermore, the extent of the presaccadic enhancement on the CSF around polar angle remains to be investigated. Presaccadic attention could benefit contrast sensitivity distinctly as a function of polar angle location: For example, because performance along the vertical meridian is worse during fixation, presaccadic attention could enhance contrast sensitivity more at these locations, where there is more room for improvement. Alternatively, polar angle asymmetries could be preserved even with the typically robust effect of presaccadic attention, as is the case for spatial (Cameron et al., 2002; Carrasco et al., 2002; Talgar et al., 2004; Purokayastha et al., 2021) and temporal attention (Fernández et al., 2019).

Here, we investigated presaccadic modulations jointly by measuring the CSF around the visual field. To investigate whether and how presaccadic attention shapes our window of visibility, we characterized the CSFs and compared the key CSF attributes (Fig. 1*b*) during fixation and saccade preparation, at the upper vertical, lower vertical, and horizontal meridians. We fit a hierarchical Bayesian model (HBM) that has been applied to analyze CSFs (Y. Zhao et al., 2021a,b), which decomposes the variability of the dataset into multiple hierarchies and takes into consideration potential relations between CSF parameters. Thus, the model enables precise estimates of the CSF parameters and metrics with relatively few observations, including peak contrast sensitivity (peak-CS), the preferred SF at the peak-CS (peak-SF), bandwidth (full width at half maximum), the highest discernable SF (cutoff-SF), and area under the log CSF (AULCSF).

We found polar angle asymmetries in peak-CS, cutoff-SF, and AULCSF during fixation (Jigo et al., 2023). Importantly, presaccadic attention (1) shifted the CSF upward, increasing the peak-CS; (2) shifted the CSF rightward to higher SFs, increasing the peak-SF and cutoff-SF; (3) narrowed the CSF, decreasing the bandwidth; and (4) increased the AULCSF. These presaccadic modulations were present at all polar angle locations, but there was a larger presaccadic enhancement along the horizontal than the vertical meridian, specifically for the peak-CS as well as for contrast sensitivity across a wide range of SFs. These findings reveal that presaccadic attention increases our window of visibility, narrowing the difference between visual representations at the peripheral saccade target and the future fovea, and more so before horizontal than vertical saccades.

## Materials and Methods

### Participants

Twelve observers (4 males and 8 females, including authors YK and NH; ages 20–34) with normal or corrected-to-normal vision participated in the experiment. All participants (except for the two authors) were naive regarding the goal of the study and were paid $12 per hour. The experimental procedures were approved by the Institutional Review Board at New York University, and all participants provided informed consent. All procedures agreed with the Declaration of Helsinki.

### Setup

Participants sat in a dark room with their head stabilized by a chin and forehead rest. All stimuli were generated and presented using MATLAB (MathWorks) and Psychophysics Toolbox (Brainard, 1997; Pelli, 1997) on a gamma-linearized 20 inch ViewSonic G220fb CRT screen at a viewing distance of 79 cm. The CRT screen had a resolution of 1,280 by 960 pixels and a refresh rate of 100 Hz. Gaze position was recorded using an EyeLink 1000 Desktop Mount eye tracker (SR Research) at a sampling rate of 1 kHz. The EyeLink Toolbox was used for eye tracking with MATLAB and Psychophysics Toolbox (Cornelissen et al., 2002).

### Experimental procedure

**Figure 2.**
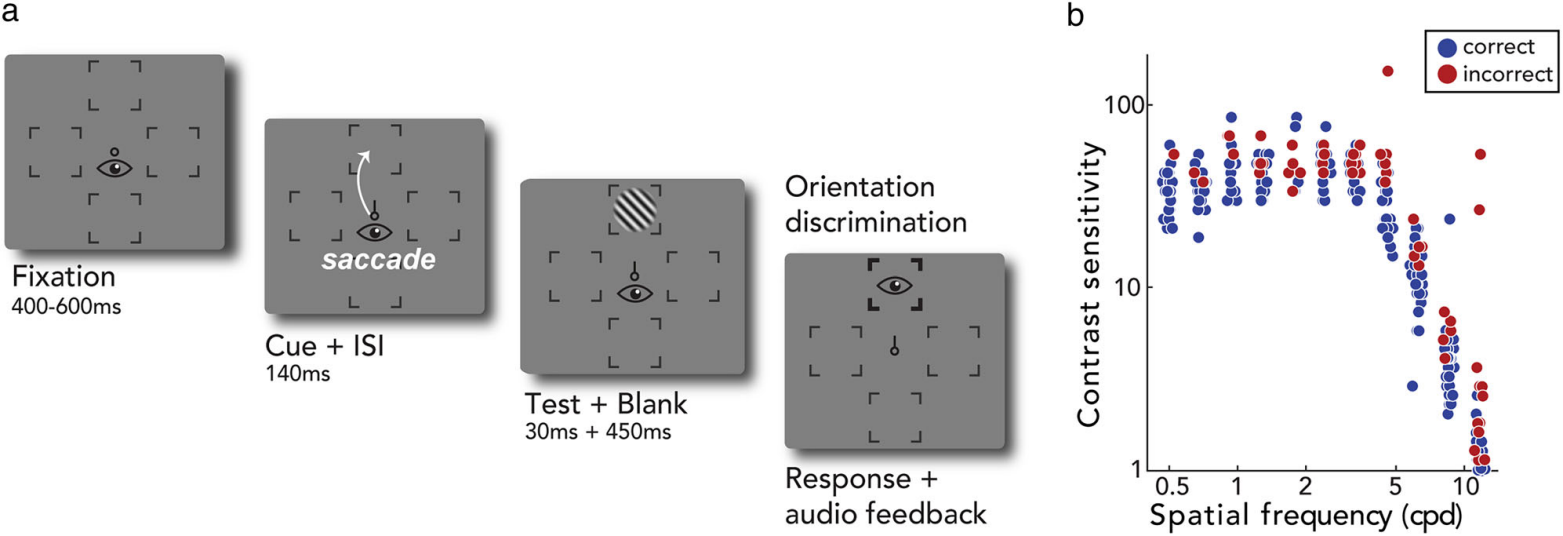
Experimental procedure. ***a***, Trial sequence. In saccade blocks (presaccadic attention), a central saccade cue indicated the saccade target after a fixation period. Participants were instructed to make a saccade precisely to the center of the corresponding placeholder as soon as the cue appeared. Just before saccade onset, a test Gabor stimulus was presented briefly at the cued location, and participants judged its orientation (CW or CCW) after response cue onset (i.e., after the saccade). The spatial frequency and the contrast of the Gabor on each trial were varied based on the qCSF (quick contrast sensitivity function) procedure. The eye icons are for illustration only and were not shown during the experiment. In the fixation blocks (not illustrated here), the trial sequence was identical except that the cue pointed to all four locations, and participants were instructed to fixate at the center throughout the entire block. ISI stands for interstimulus interval. ***b***, Trial-by-trial data points for an example participant and location. Blue and red dots indicate trials with correct and incorrect responses, respectively. The qCSF procedure efficiently samples stimuli in spatial frequency and contrast space based on responses on previous trials. For individual data see Extended Data Figure 2-1.

Participants performed an orientation discrimination task for stimuli varying in contrast and spatial frequency, during fixation and saccade preparation (presaccadic attention; Fig. 2). To place trials efficiently in the dynamic range of the CSF, we leveraged the qCSF (quantitative contrast sensitivity function) method for data collection (Lesmes et al., 2010). Given participants’ performance in previous trials, this procedure selects stimulus contrast and spatial frequency to be tested on the next trial, based on the maximum expected information gain, to further refine the CSF parameter estimates. The possible stimulus space was composed of 60 contrast levels from 0.001 to 1, and 12 spatial frequency levels from 0.5 to 16 cpd, evenly spaced in log units.

10.1523/ENEURO.0243-24.2024.f2-1 Figure 2-1 **Individual observer data.** The trial-by-trial datapoints and the model fits are shown for four observers, separately for each condition. Blue and red datapoints indicate trials with correct and incorrect responses, and the solid black line is the fitted HBM. Download Figure 2-1, TIF file.

We manipulated eye movement instruction (fixation and saccade preparation, from now on “instruction”) between experimental blocks. Figure 2*a* shows the trial sequence under the saccade instruction. Each trial started with a fixation circle (0.175° radius) on a gray background (∼26 cd/m^2^) with a duration randomly jittered between 400 and 600 ms. Four placeholders indicated the locations of the upcoming stimuli, 6° left, right, above, and below fixation. Measurements at the left and right locations were combined for analysis, as it is well established that contrast sensitivity with binocular viewing does not differ between these locations (Cameron et al., 2002; Himmelberg et al., 2020). Each placeholder was composed of four corners (black lines, 0.2° length). The trial began once a 300 ms stable fixation (eye coordinates within a 1.75° radius virtual circle centered on fixation) was detected.

In blocks with the saccade instruction (saccade preparation), after the fixation period, a central cue (black line, 0.35° length) pointed to one of the four cardinal placeholders, indicating the saccade target; the cue direction was randomly interleaved. Participants were instructed to move their eyes to the center of the cued location as fast and precisely as possible. At 140 ms after cue onset (i.e., before saccade onset), a test Gabor grating (tilted ±45 relative to vertical, random phase, delimited by a raised cosine envelope with 2° radius) appeared for 30 ms at the cued saccade target location. The spatial frequency and the contrast of the Gabor stimulus were determined by the qCSF procedure on each trial (Lesmes et al., 2010), based on the maximum expected information gain (reduction in entropy). At 450 ms after stimulus offset (and after saccade offset), the cued location was highlighted by increasing the width and length of the corresponding placeholders. Participants performed an un-speeded, orientation discrimination task for the Gabor grating at the cued location (by pressing left arrow key for −45°, right arrow key for +45°).

Stimulus parameters and timing for the blocks with the fixation instruction were identical to the saccade blocks with one exception: Instead of one saccade target cue, four black direction cue lines pointed to all locations. Participants maintained fixation throughout the entire trial sequence. The baseline condition, with spatially noninformative cues indicating all possible target locations, has been used in studies investigating presaccadic attention (Rolfs and Carrasco, 2012; Li et al., 2016, 2019; Hanning et al., 2022, 2023, 2024), as well as covert attention (Talgar et al., 2004; Jigo and Carrasco, 2020; Fernández et al., 2022). The studies that have compared spatially noninformative and informative cues found no difference between the two (Rolfs and Carrasco, 2012; Li et al., 2016, 2019). Only with a sufficiently long interstimulus interval (400 ms) to deploy endogenous covert attention, a spatially informative cue yields significant enhancements in the contrast response function (Li et al., 2021b). In any case, we tested some participants in a control condition (*n* = 5), in which all stimulus parameters were identical, and a spatially informative cue was presented but the saccade was not performed. The interstimulus interval of 140 ms was too short for covert endogenous attention to be deployed (review, see Carrasco, 2011).

Gaze position was monitored online to ensure fixation within a 1.75° radius virtual circle from the central fixation until the response phase (fixation blocks) or until cue onset (saccade blocks). Trials in which gaze deviated from fixation were aborted and repeated at the end of each block. In addition, in the saccade blocks, we repeated trials in which saccade latency was too short (<150 ms) or too long (>350 ms) or in which saccades landed outside of a 2.25° radius virtual circle around the saccade target center. Moreover, because we are interested in the presaccadic interval, we only included trials in which saccades were initiated after stimulus offset.

### CSF analysis

#### Bayesian inference

**Figure 3.**
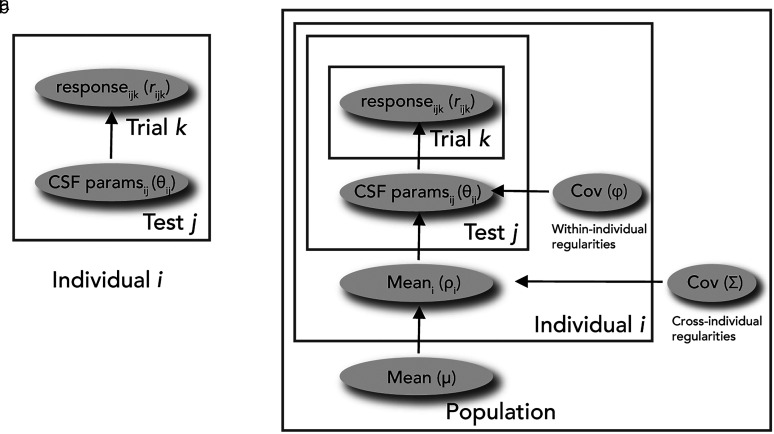
CSF model fitting. ***a***, Schematic representation of the BIP (Bayesian inference procedure). The first stage of the data analysis consists of fitting trial-by-trial data points with the BIP to estimate the CSF parameters—peak contrast sensitivity (peak-CS), peak spatial frequency (peak-SF), and bandwidth. The BIP computes the posterior distribution of the parameters for each test independently. ***b***, Schematic representation of the HBM (hierarchical Bayesian model). In the second stage of the data analysis, the BIP outputs are used as the mean and covariance of the prior distributions of the CSF hyperparameters in the HBM. We used a three-level hierarchical model to incorporate potential relations in the CSF parameters across individuals and tests. For details on the BIP and the HBM, see CSF analysis.

To characterize the CSFs during fixation and saccade preparation for each of the four locations, we used the Bayesian inference procedure (BIP; Fig. 3*a*) and the hierarchical Bayesian model (HBM; Fig. 3*b*) to fit the three-parameter CSF model to the trial-by-trial data (Fig. 2*b*; Y. Zhao et al., 2021a,b). For both the BIP and the HBM, Bayesian inference was used to estimate the posterior distribution of the three CSF parameters—peak contrast sensitivity (peak-CS), peak spatial frequency (peak-SF), and bandwidth. Other key attributes, such as the cutoff spatial frequency (cutoff-SF) and area under the log CSF (AULCSF), were computed from the final estimate of the CSF. For individual data, see Extended Data Figure 2-1.

Contrast sensitivity S at spatial frequency sf is modeled as a log-parabola function with parameters θ=(peakCS,peakSF,bandwidth) (Watson and Ahumada, 2005; Lesmes et al., 2010):

$$\log_{10}\;(S(\mathrm{sf},\theta ))\;=\;\log_{10}\;(\mathrm{peakCS})-\frac{4}{\log_{10}(2)}{\left(\frac{\log_{10}(\mathrm{sf})-\log_{10}(\mathrm{peakSF})}{\mathrm{bandwidth}}\right)}^{2}.$$

The probability of a correct response (r=1) on a trial given the stimulus—spatial frequency sf and contrast c—is described as a psychometric function:

$$p\;(r=1\;|\theta ,\;\mathrm{sf},c)\;=\;g\;+\;(1-g-\lambda /2)\;\left(1-\exp\;\left(-{\left(\mathrm{cS}\left(\mathrm{sf},\theta \right)\right)}^{\beta }\right)\right),$$

where g is the guessing rate (g=0.5), λ is the lapse rate ( λ=0.04; Wichmann and Hill, 2001; Y. Zhao et al., 2021b), and β determines the slope of the psychometric function (β=2). The probability of making an incorrect response (r=0) is shown in the following:

p(r=0|θ,sf,c)=1−p(r=1|θ,sf,c).

Equations 2 and 3 define the likelihood function or the probability of a correct/incorrect response in a trial given the stimulus and the CSF parameters. To infer the CSF parameters given the experimental data, Bayes’ rule (Eq. 10) is used to estimate the posterior distribution of the CSF parameters (θ) based on the likelihood and the prior obtained from the BIP procedure (Eqs. 4–6).

#### HBM: modeling prior distributions

For fitting the HBM, we start with the prior distributions of μ (population mean), $\Sigma^{-1}$ (inverse of population level covariance matrix), and $\varphi^{-1}$ (inverse of individual level covariance matrix), which are $p_{0}(\mu )$, $p_{0}(\Sigma^{-1})$, and $p_{0}(\varphi^{-1})$ respectively.

For each of the CSF parameter, the prior distribution of μ is a truncated normal distribution: N(mean, standarddeviation)T(lowerbound,upperbound). The mean and standard deviation of the truncated normal distribution is set to the average of the mean $\left(\mu_{\mathrm{peakCS},0},\mu_{\mathrm{peakSF},0},\;\mu_{\mathrm{bandwidth},0}\right)$ and standard deviation $\left(\sigma_{\mathrm{peakCS},0},\sigma_{\mathrm{peakSF},0},\sigma_{\mathrm{bandwidth},0}\right)$ of the corresponding parameters obtained from the BIP across individuals ( I=72; combination of a participant, location, and instruction):

$$p_{0}(\mu_{\mathrm{peakCS}})\;=\;N(\mu_{\mathrm{peakCS},0},\sigma_{\mathrm{peakCS},0})T\left(\log_{10}(1.05),\log_{10}(1050)\right),$$

$$p_{0}(\mu_{\mathrm{peakSF}})\;=\;N(\mu_{\mathrm{peakSF},0},\sigma_{\mathrm{peakSF},0})T\left(\log_{10}(0.1),\log_{10}(5)\right),$$

$$p_{0}(\mu_{\mathrm{bandwidth}})\;=\;N(\mu_{\mathrm{bandwidth},0},\sigma_{\mathrm{bandwidth},0})T\left(\mathrm{lo}g_{10}(1),\mathrm{lo}g_{10}(9)\right).$$

The prior distributions of the precision matrices (the inverse of covariance matrices $\Sigma^{-1}$ and $\varphi^{-1}$) are modeled as Wishart distributions. W(Y,v) denotes a Wishart distribution with expected precision matrix *Y* and degrees of freedom V(v=4). $\Sigma_{\mathrm{BIP}}$ and $\varphi_{\mathrm{BIP}}$ are population-level and individual-level covariance matrices obtained from the BIP:

$$p_{0}(\Sigma^{-1})=W\left(\frac{\Sigma_{\mathrm{BIP}}^{-1}}{v},\;v\right),$$

$$p_{0}(\varphi^{-1})=W\left(\frac{\varphi_{\mathrm{BIP}}^{-1}}{v},\;v\right).$$

#### HBM: three-level hierarchy

We used a simple three-level HBM without considering any structure related to the experimental conditions, as reported in Y. Zhao et al. (2021a; Fig. 3*b*). In the current study, an individual refers to a combination of a participant, location (upper, lower, horizontal), and instruction (fixation, saccade preparation) condition (*I *= 72). In addition, all trials that each participant completed were combined into one test (*J *= 1).

The BIP fits these parameters independently for each individual and test (Fig. 3*a*). Although the BIP has been proven to be a good estimate of the CSF, it may have overestimated the variance of each test because it scores each test independently with a uniform prior without considering potential relations of the parameters (Y. Zhao et al., 2021a). Therefore, we used the hierarchical model which recently has been shown to reduce the uncertainties of the parameter estimates when fitting the CSF (Y. Zhao et al., 2021a,b). The HBM considers potential relations of the CSF parameters and hyperparameters within and across individuals (Fig. 3*b*). More specifically, it quantifies the joint distribution of the CSF parameters and hyperparameters at three hierarchies in a single model: test (*J*), individual (*I*), and population level. The within-individual and cross-individual regularities are modeled with covariances of the CSF hyperparameters at the individual (φ) and population levels (Σ), respectively. Incorporating this knowledge into the model and decomposing the variability of the entire dataset into distributions at multiple hierarchies enabled us to reduce the variance of the test-level estimates and to obtain more precise estimates of the CSF parameters.

We fit the BIP and the HBM sequentially. As a first step, the BIP was fit to obtain the mean and standard deviation of each of the three CSF parameters across all participants and conditions, using a uniform prior distribution. Next, these values were fed into the HBM and set the mean and covariance of the prior distributions of the CSF hyperparameters (Eq. 4).

At the population level of the HBM, the joint distribution of hyperparameter η was modeled as a mixture of three-dimensional Gaussian distributions N with mean μ and covariance Σ, which have distributions p(μ) and p(Σ):

p(η)=N(η,μ,Σ)p(μ)p(Σ).

At the individual level (I), the joint distribution of hyperparameter $\tau_{i}$ of individual i was modeled as a mixture of three-dimensional Gaussian distributions N with mean $\rho_{i}$ and covariance φ, which have distributions $p(\rho_{i}|\eta )$ and p(φ), where $p(\rho_{i}|\eta )$ denotes that the mean $\rho_{i}$ was conditioned on the population-level hyperparameter η:

$$p(\tau_{i}|\eta )\;=\;N(\tau_{i},\;\rho_{i},\varphi )p(\rho_{i}|\eta )p(\varphi ).$$

At the test level (J), $p(\theta_{ij}|\tau_{i})$, the joint distribution of parameter $\theta_{ij}$ of individual i and test j was conditioned on hyperparameter $\tau_{i}$ at the individual level.

The probability of obtaining the entire dataset was computed by probability multiplication:

$$\begin{array}{rl} \;p(r_{1:I,1:J,1:K}|X) & =\prod_{i=1}^{I}\prod_{\;j=1}^{J}\;p(r_{ij,1:K}|\theta_{ij})\;p(\theta_{ij}|\tau_{i})\;p(\tau_{i}|\eta )\;p(\eta )\;\\ & =\prod_{i=1}^{I}\prod_{\;j=1}^{J}\;p(r_{ij,1:K}|\theta_{ij})\;p(\theta_{ij}|\tau_{i})\;N(\tau_{i},\rho_{i},\varphi )\;p(\rho_{i}|\eta )\;p(\varphi )\;N(\eta ,\;\mu ,\;\Sigma )\;p(\mu )\;p(\Sigma ), \end{array}$$

where $X=(\theta_{1:I,1:J},\rho_{1:i},\;\varphi ,\mu ,\Sigma )$ are all parameters and hyperparameters in the HBM, I is the total number of individuals, and J is the total number of tests on each individual.

#### HBM: computing the joint posterior distribution

Bayes’ rule $\left(\mathrm{posterior}\;\mathrm{probability}\;p(A|B)=\frac{\mathrm{likelihood}\;p(B|A)\;\times \;\mathrm{prior}\;\mathrm{probability}\;p(A)}{\mathrm{marginal}\;\mathrm{probability}\;p(B)}\right)$ was used to compute the joint posterior distribution of all the parameters and hyperparameters in the HBM:

$$p(X|r_{1:I,1:J,1:K})=\frac{\prod_{I=1}^{I}\prod_{J=1}^{J}\;p(r_{ij,1:K}|\theta_{ij})\;p(\theta_{ij}|\tau_{i})\;N(\tau_{i},\rho_{i},\varphi )\;p(\rho_{i}|\eta )\;p_{0}(\varphi )\;N(\eta ,\mu ,\Sigma )\;p_{0}(\mu )\;p_{0}(\Sigma )}{\int \prod_{I=1}^{I}\prod_{J=1}^{J}\;p(r_{ij,1:K}|\theta_{ij})\;p(\theta_{ij}|\tau_{i})\;N(\tau_{i},\rho_{i},\varphi )\;p(\rho_{i}|\eta )\;p_{0}(\varphi )\;N(\eta ,\mu ,\Sigma )\;p_{0}(\mu )\;p_{0}(\Sigma )\;dX},$$

where the denominator is the probability of obtaining the entire dataset (Eq. 9).

We used the JAGS package (Plummer, 2003) in R (R Core Team, 2020) to evaluate the joint posterior distribution. JAGS generates representative samples of the joint posterior distribution of all the parameters and hyperparameters in the HBM via Markov chain Monte Carlo (MCMC). We ran three parallel MCMC chains, each generating 2,000 samples, resulting in a total of 6,000 samples. Steps in the burn-in and adaptation phases—20,000 and 500,000 steps, respectively—were discarded and excluded from the analysis because the initial part of the random walk process is largely determined by random starting values. The code for the HBM analysis will be available upon reasonable request.

### Eye movement analysis

In addition to the gaze monitoring and detection of saccades online (see above, Experimental procedure), we also performed an offline eye movement analysis using an established algorithm for saccade detection (Engbert and Mergenthaler, 2006). Saccades were detected based on their velocity distribution using a moving average over 20 subsequent eye position samples. Saccade onsets and offsets were detected based on when the velocity exceeded or fell below the median of the moving average by 3 standard deviations for at least 20 ms. This offline analysis considers the entire distribution of eye position samples and velocity and allows for a more detailed evaluation of both fixation and saccadic eye movements. In total, we included 22,527 trials in the analysis—on average 1,877 ± 55 (mean ± 1 SEM) trials per observer.

### Statistical analysis

Convergence of the HBM parameters was determined based on Gelman and Rubin's diagnostic rule (Gelman and Rubin, 1992). Each parameter and hyperparameter were considered to have “converged” when the variance of the samples across the MCMC chains divided by the variance of the samples within each chain was smaller than 1.05. In the current study, all parameters of the HBM converged.

To obtain final estimates of the CSF parameters in the HBM—peak-CS, peak-SF, and bandwidth—parameter estimates of the 6,000 samples were averaged for each participant and location–instruction combination. The final CSFs for each participant and location–instruction combination were obtained by inputting the final parameter estimates to Equation 1. From these final CSFs, cutoff-SF and AULCSF were computed. These key CSF attribute values were used for statistical testing.

All *p* values reported are based on permutation testing over 1,000 iterations with shuffled data. *p* value is the proportion of a metric (*F* score or *t* score) in the permuted null distribution greater than or equal to the metric computed using intact data. Note that the minimum *p* value achievable with this procedure is 0.001. *p* values were FDR corrected for multiple comparisons, when applicable (Benjamini and Hochberg, 1995).

We also performed Bayesian statistics using the “BayesFactor” package in R (version 0.9.12-4.4). We report BF_10_ only for null or marginal results from permutation testing (*p *> 0.05). The Bayes factors (BF_10_) for main effects and interaction effects in the repeated-measures ANOVA design were computed by comparing the full model (H1) against the restricted model (H0) in which the effect of interest was excluded from the full model (Rouder et al., 2017). BF_10_ smaller than 1 is in support for the absence of an effect; values 1–3, 3–10, 10–30, 30–100, and >100 indicate anecdotal, moderate, strong, very strong, and extreme evidence for the presence of an effect (Kass and Raftery, 1995; Lee and Wagenmakers, 2014).

To evaluate the goodness-of-fit of the trial-by-trial data points to the fitted CSF, we compared the maximum likelihood value of the reported model against a null model assuming a flat CSF. For the null model, contrast sensitivity was set as the contrast sensitivity averaged across SFs. The negative log-likelihood values were −147.83 and −216.84, respectively, for the reported and the null model, indicating a better fit for the reported model. Likelihood ratio test using a Chi-square distribution with the degrees of freedom = 453 (full model)—72 (null model) indicated a significant difference between the two models (*p *< 0.001).

## Results

**Figure 4.**
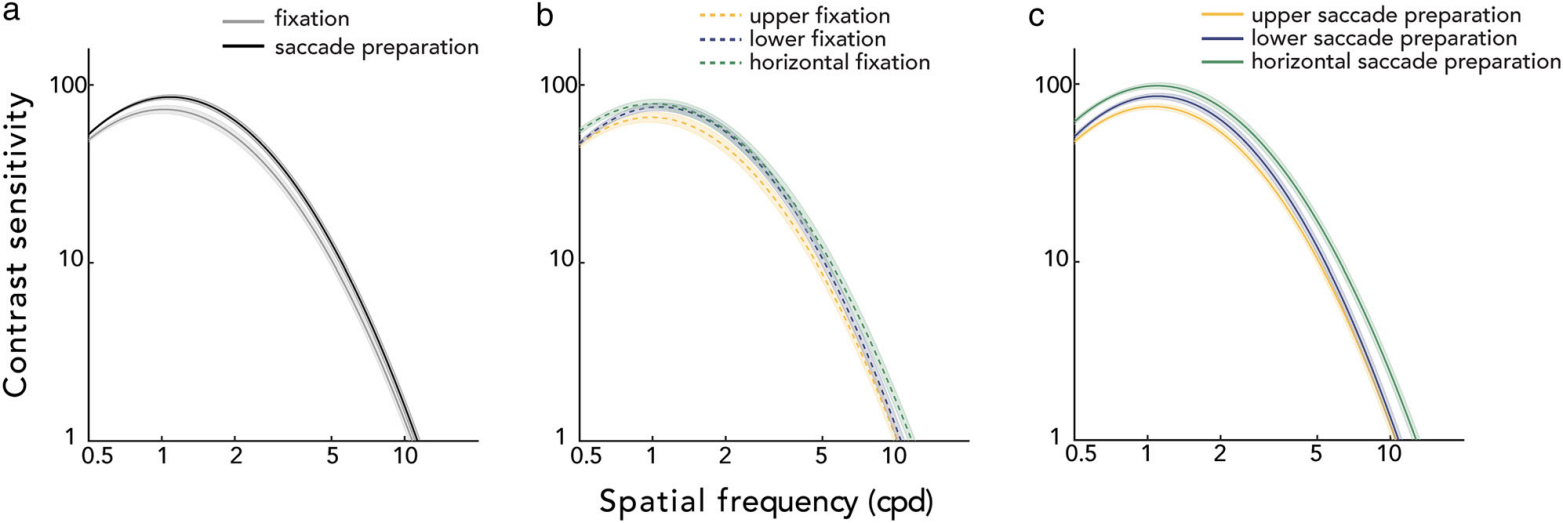
Group average CSFs. ***a***, CSFs for the fixation and saccade preparation instruction, collapsed across locations. Saccade preparation modulates contrast sensitivity across spatial frequencies. ***b***, CSFs during fixation for each location, exhibiting polar angle asymmetries. ***c***, CSFs during saccade preparation, separately for each location. Polar angle asymmetries in the CSFs during fixation were also present during saccade preparation. ***a–c***, The CSF parameters were fitted with the HBM (hierarchical Bayesian model). For a demo of the presaccadic attention effects on the CSF, see Extended Data Figure 4-1. Shaded regions denote ±1 standard error of the mean (SEM).

Figure 4 shows the CSFs averaged across participants, during fixation and saccade preparation (presaccadic attention) for each location. To preview the results, saccade preparation shifted and reshaped the CSFs during fixation for all locations, increasing overall contrast sensitivity (Fig. 4*a*). Second, during fixation (Fig. 4*b*), contrast sensitivity was better in the horizontal than the vertical meridian, and in the lower vertical than the upper vertical meridian, consistent with Horizontal-Vertical Anisotropy (HVA) and Vertical Meridian Asymmetry (VMA). Third, this pattern was overall maintained during saccade preparation (Fig. 4*c*), but the presaccadic benefit on contrast sensitivity was more pronounced at the horizontal than at the vertical meridian across a wide spatial frequency range.

10.1523/ENEURO.0243-24.2024.f4-1 Figure 4-1 **Demo of presaccadic effects on the CSF.** A Landolt-C stimulus image was filtered with the group-average CSF parameters in the fixation and saccade preparation conditions. The demo changes from the perceived image during fixation to during saccade preparation, both at the horizontal location. *(The demo was submitted separately as a .mov file)* Download Figure 4-1, TIF file.

10.1523/ENEURO.0243-24.2024.v1 Figure 4-1. Download Figure 4-1., MP4 file.

**Figure 5.**
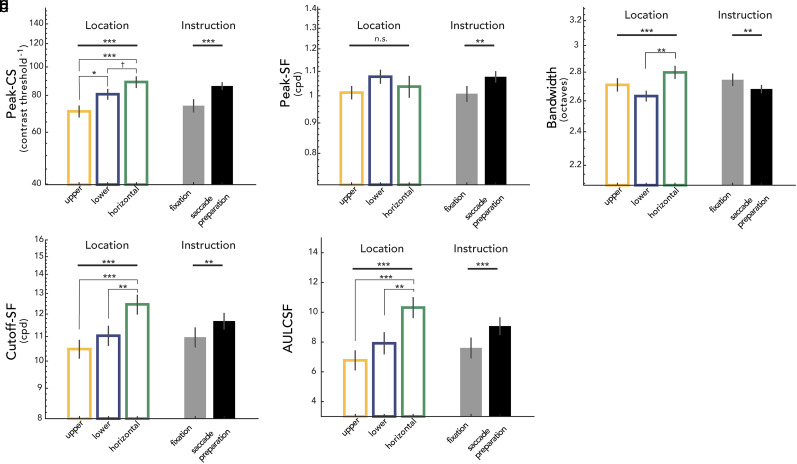
Main effects of location (averaged across fixation and saccade preparation instruction) and eye movement instruction (averaged across locations). ***a***, peak-CS; ***b***, peak-SF; ***c***, bandwidth; ***d***, cutoff-SF; ***e***, AULCSF. Error bars indicate ±1 standard error of the mean (SEM). Main effects of location and instruction are shown above the thick gray horizontal bars on the top. For a control condition (fixation + spatially informative cue), see text and Extended Data Figure 5-1. Significant post hoc pairwise comparisons of location effects are denoted by asterisks above thin gray bars. ***p < 0.001, **p < 0.01, *p < 0.05, ^†^*p *< 0.1, n.s., not significant; FDR corrected.

We conducted a two-way repeated-measures ANOVA for each of the key CSF attributes, with location (upper, lower, horizontal) and eye movement instruction (fixation, saccade preparation) as within-subject factors (Fig. 5).

10.1523/ENEURO.0243-24.2024.f5-1 Figure 5-1 **Results for the control condition.** The control condition, in which participants (n = 5) maintained fixation while a spatially informative cue was presented, was compared with the fixation condition with a non-informative cue in the main study. The CSF and its key attributes did not differ between the two conditions. **(a)** Group average CSFs. **(b-f)** Group average key CSF attributes: **(b)** peak-CS; **(c)** peak-SF; **(d)** bandwidth; **(e)** cutoff-SF; **(f)** AULCSF. Error bars indicate ±1 standard error of the mean (SEM). Download Figure 5-1, TIF file.

### Peak-CS: peak contrast sensitivity

**Figure 6.**
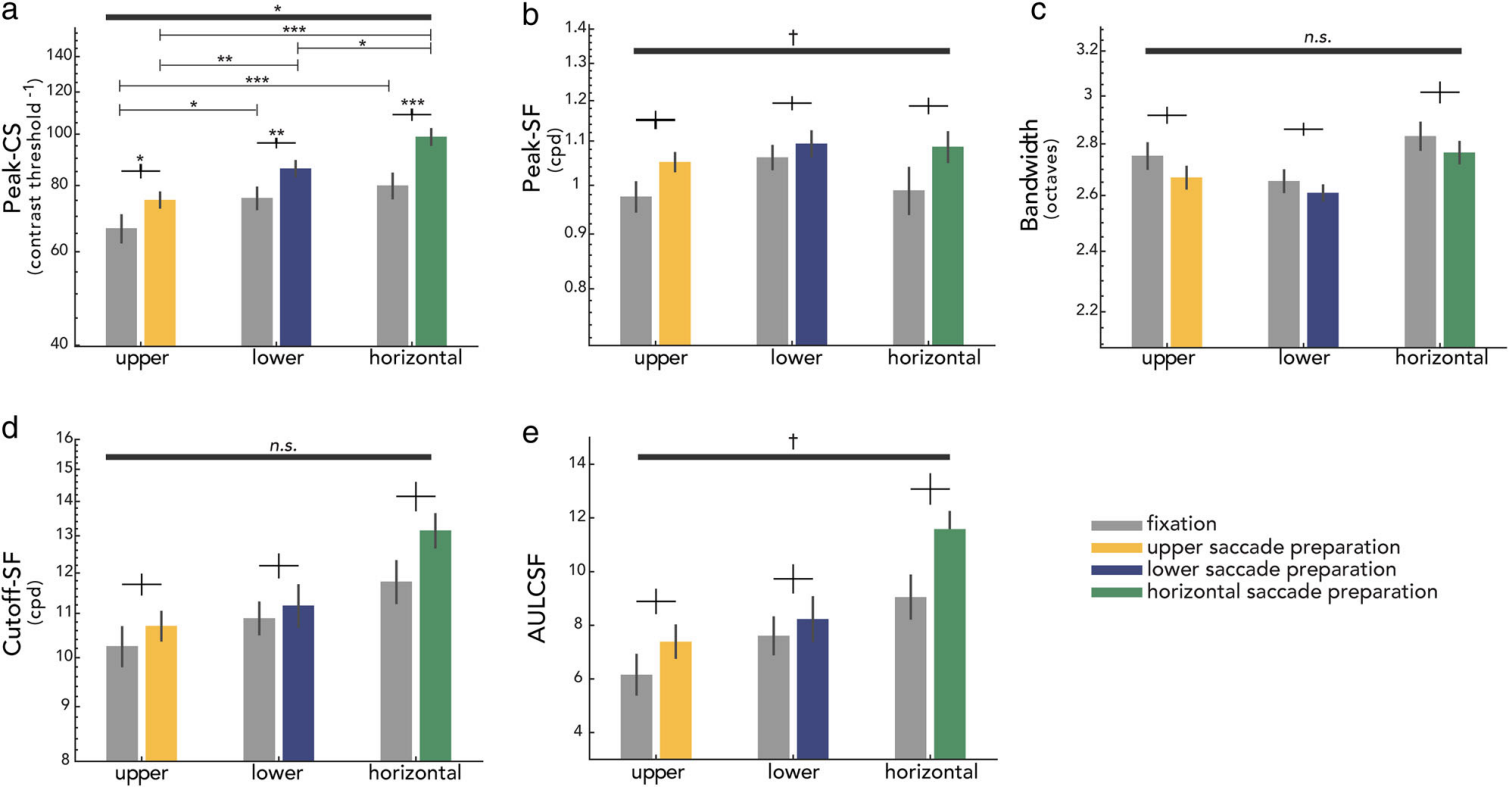
Group average key CSF attributes for each location and eye movement instruction. ***a***, peak-CS; ***b***, peak-SF; ***c***, bandwidth; ***d***, cutoff-SF; ***e***, AULCSF. Error bars indicate ±1 standard error of the mean (SEM), and vertical bars depict ±1 standard error of the difference (SED). Location–instruction interaction effects are shown at the top of each figure with dark gray horizontal bars. Significant post hoc pairwise comparisons of location and instruction effects are denoted by asterisks above black crosses and horizontal lines, respectively. ***p < 0.001, **p < 0.01, *p < 0.05, ^†^*p *< 0.1, n.s., not significant, FDR corrected.

We observed the main effects of location (*F*_(2,22)_ = 18.266; *p *< 0.001; *η*^2^ = 0.624) and instruction (*F*_(1,11)_ = 26.311; *p *< 0.001; *η*^2^ = 0.705) and an interaction effect (*F*_(2,22)_ = 18.266; *p *= 0.014; *η*^2^ = 0.322) on peak-CS (Figs. 5*a*, 6*a*). As can be seen in Figure 6*a*, there were significant location effects during fixation (*F*_(2,22)_ = 9.356; *p *= 0.003; *η*^2^ = 0.460), as well as saccade preparation (*F*_(2,22)_ = 18.266; *p *< 0.001; *η*^2^ = 0.704). During fixation, peak-CS was significantly higher for the horizontal (*t*_(11)_ = 6.137; *p *< 0.001; *d *= 0.903) and for the lower vertical meridian (*t*_(11)_ = 2.655; *p *= 0.025; *d *= 0.678) than the upper vertical meridian. With saccade preparation, peak-CS was still higher for the horizontal (*t*_(11)_ = 8.486; *p *< 0.001; *d *= 2.118) and the lower vertical (*t*_(11)_ = 3.797; *p *= 0.005; *d *= 1.089) than the upper vertical meridian, similar to the results during fixation. In addition, peak-CS became higher for the horizontal than the lower vertical meridian (*t*_(11)_ = 3.096; *p *= 0.013; *d *= 1.034), unlike the results during fixation.

Saccade preparation increased peak-CS compared with fixation for all locations (Fig. 6*a*)—upper vertical (*t*_(11)_ = 3.159; *p *= 0.013; *d *= 0.711), lower vertical (*t*_(11)_ = 4.534; *p *= 0.002; *d *= 0.844), and horizontal (*t*_(11)_ = 5.824; *p *< 0.001; *d *= 1.245) meridian, but the effect was significantly larger at the horizontal meridian than at the upper vertical meridian (*t*_(11)_ = 2.833; *p *= 0.049; *d *= 0.158) and marginally larger than at the lower (*t*_(11)_ = 2.370; *p *= 0.056; *d *= 0.151; BF_10 _= 2.083) vertical meridian. In summary, when compared with fixation, saccade preparation shifts the CSF upward and increases contrast sensitivity, for all locations, but more so for the horizontal meridian.

### Peak-SF: spatial frequency leading to the peak-CS

There was a main effect of instruction (*F*_(1,11)_ = 16.355; *p *= 0.001; *η*^2^ = 0.598), but not a main effect of location (*F*_(2,22)_ = 1.756; *p *= 0.196; *η*^2^ = 0.138; BF_10 _= 1.454) on peak-SF (Fig. 5*b*). The location–instruction interaction was marginally significant (*F*_(2,22)_ = 3.303; *p *= 0.057; *η*^2^ = 0.231; BF_10 _= 0.335), resulting from presaccadic enhancement (increased peak-SF during saccade preparation relative to fixation) surviving FDR correction only for the horizontal (*t *= 3.082; *p *= 0.031; *d *= 0.728) and the upper vertical (*t *= 3.082; *p *= 0.031; *d *= 0.728) meridian (Fig. 6*b*). Taken together, (1) peak-SF during fixation did not differ across locations; (2) saccade preparation increased the peak-SF compared with fixation, resulting in a rightward shift of the CSF; and (3) the effect of saccade preparation on peak-SF was present at all locations. These results show that during saccade preparation, contrast sensitivity is enhanced around the visual field particularly for spatial frequencies above the fixation peak-SF.

### Bandwidth: full width at half maximum

There were main effects of location (*F*_(2,22)_ = 7.137; *p *< 0.001; *η*^2^ = 0.396) and instruction (*F*_(1,11)_ = 10.258; *p *= 0.005; *η*^2^ = 0.483) and no significant interaction (*F*_(2,22)_ = 0.289; *p *= 0.752; *η*^2^ = 0.026; BF_10 _= 0.220) on bandwidth (Figs. 5*c*, 6*c*). To further examine the main effect of location, bandwidth values were averaged across fixation and saccade preparation within each location for comparison across location pairs (Fig. 5*c*). Only the difference between the lower vertical and the horizontal meridian survived FDR correction: The bandwidth at the lower vertical meridian was narrower than at the horizontal meridian (*t*_(11)_ = 4.142; *p *= 0.005; *d *= 1.134). The main effect of instruction reflects the bandwidth narrowing with saccade preparation (Fig. 5*c*), and the lack of location–instruction interaction indicates that this effect is similar around the visual field (Fig. 6*c*).

### Cutoff-SF: highest perceivable spatial frequency at which contrast sensitivity is 1.0

We observed the main effects of location (*F*_(2,22)_ = 22.232; *p *< 0.001; *η*^2^ = 0.669) and instruction (*F*_(1,11)_ = 18.250; *p *= 0.002; *η*^2^ = 0.624), and no significant location–instruction interaction (*F*_(2,22)_ = 2.285; *p *= 0.125; *η*^2^ = 0.172; BF_10 _= 0.860) on cutoff-SF (Figs. 5*d*, 6*d*). To follow up the location effect, we conducted post hoc comparisons for location pairs on cutoff-SF values averaged across fixation and saccade preparation for each location (Fig. 5*d*). The cutoff-SF at the horizontal meridian was significantly higher than that at the upper (*t*_(11)_ = 7.787; *p *< 0.001; *d *= 1.324) and the lower vertical meridian (*t*_(11)_ = 4.181; *p *= 0.002; *d *= 0.902). The main effect of instruction was caused by saccade preparation increasing the cutoff-SF (Fig. 5*d*). This presaccadic enhancement on cutoff-SF was similar around the visual field (Fig. 6*d*). These results, together with the increase in peak-SF, indicate that saccade preparation shifts the CSF rightward and enhances contrast sensitivity more for spatial frequencies higher than peak-SF during fixation.

### AULCSF: area under the log CSF

We observed the main effects of location (*F*_(2,22)_ = 21.626; *p *< 0.001; *η*^2^ = 0.663) and instruction (*F*_(1,11)_ = 26.642; *p *< 0.001; *η*^2^ = 0.708) on AULCSF (Fig. 5*e*). The location–instruction interaction was marginally significant (*F*_(2,22)_ = 3.109; *p*= 0.070; *η*^2^ = 0.220; BF_10 _= 0.207), from presaccadic enhancements in AULCSF surviving FDR correction only at the horizontal (*t*(11) = 4.365; *p *= 0.003; *d *= 0.961) and the upper vertical (*t*(11) = 2.602; *p *= 0.040; *d *= 0.500) meridian (Fig. 6*e*). Post hoc analyses of location effects were conducted on AULCSF values averaged across fixation and saccade preparation for each location (Fig. 5*e*): AULCSF was significantly larger for the horizontal than the upper (*t*_(11)_ = 8.725; *p *< 0.001; *d *= 1.486) and lower (*t*_(11)_ = 3.900; *p *= 0.003; *d *= 0.957) vertical meridian. Thus, saccade preparation increases AULCSF at all locations, widening the window of visibility and rendering more of the world perceivable at the saccade target around the visual field.

### Presaccadic benefit on contrast sensitivity across spatial frequencies

**Figure 7.**
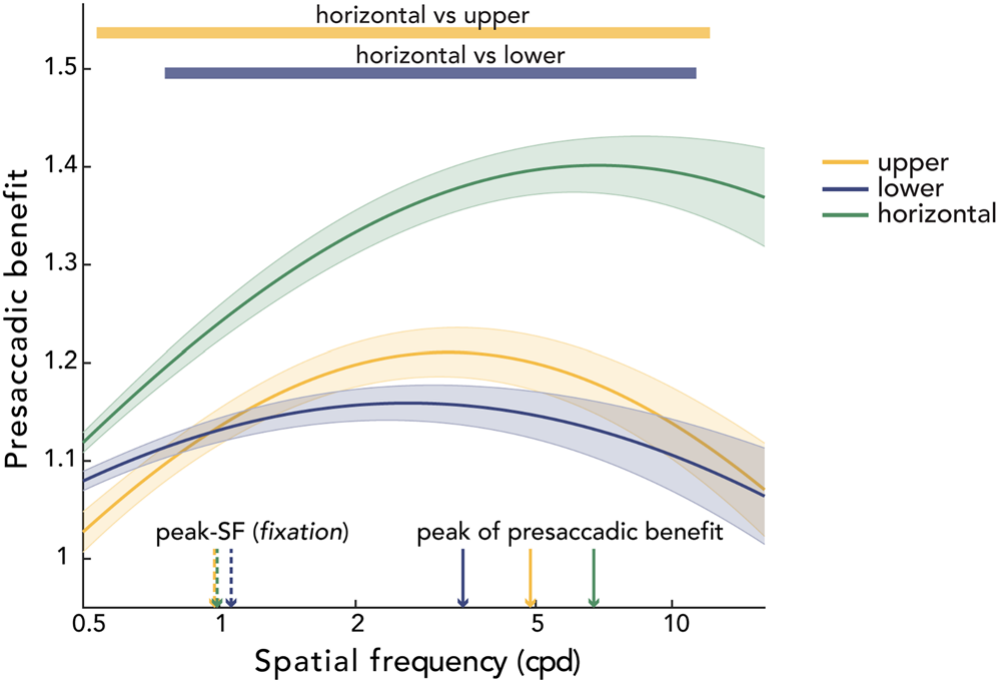
Presaccadic benefit, quantified as the ratio of contrast sensitivity during saccade preparation and fixation. Group average presaccadic benefit on the CSF, separately for each location. The yellow and blue horizontal bars at the top mark the spatial frequencies at which horizontal enhancement was larger than at the upper and lower vertical meridian, respectively. The vertical dashed arrows denote group peak-SF during fixation separately for each location. The vertical solid arrows indicate the spatial frequency resulting in the peak of the presaccadic benefit for each location. Note that the vertical solid lines do not necessarily correspond to the spatial frequency resulting in the maximum value of the group averaged curve shown in the figure. Shaded regions represent ±1 standard error of the mean (SEM).

Saccade preparation improved performance across a wide range of spatial frequencies. There were main effects of location (*F*_(2,22)_ = 7.162; *p *= 0.003; *η*^2^ = 0.394) and spatial frequency (*F*_(50,550)_ = 9.957; *p *< 0.001; *η*^2^ = 0.143) and a significant interaction effect (*F*_(100,1100)_ = 1.841; *p *< 0.001; *η*^2^ = 0.672) on the magnitude of the presaccadic benefit. To better understand the interaction effect, we conducted a one-way repeated-measures ANOVA with location as a factor for each spatial frequency and observed a significant location effect in a wide spatial frequency range (Fig. 7; between 0.616 and 11.314 cpd; *p *< 0.05). The presaccadic benefit at the horizontal meridian was greater than at the upper vertical meridian (between 0.536 and 12.126 cpd; *p *< 0.05) and the lower vertical meridian (between 0.758 and 11.314 cpd; *p *< 0.05). The presaccadic benefit did not differ between the upper and lower vertical meridian for any of the spatial frequencies tested. Moreover, there was a maximum benefit of saccade preparation (relative to fixation) on contrast sensitivity for spatial frequencies higher than the fixation peak-SF (Fig. 7), which is consistent with presaccadic attention shifting the peak-SF to higher spatial frequencies (Figs. 5*b*, 6*b*).

### Eye movement parameters

**Figure 8.**
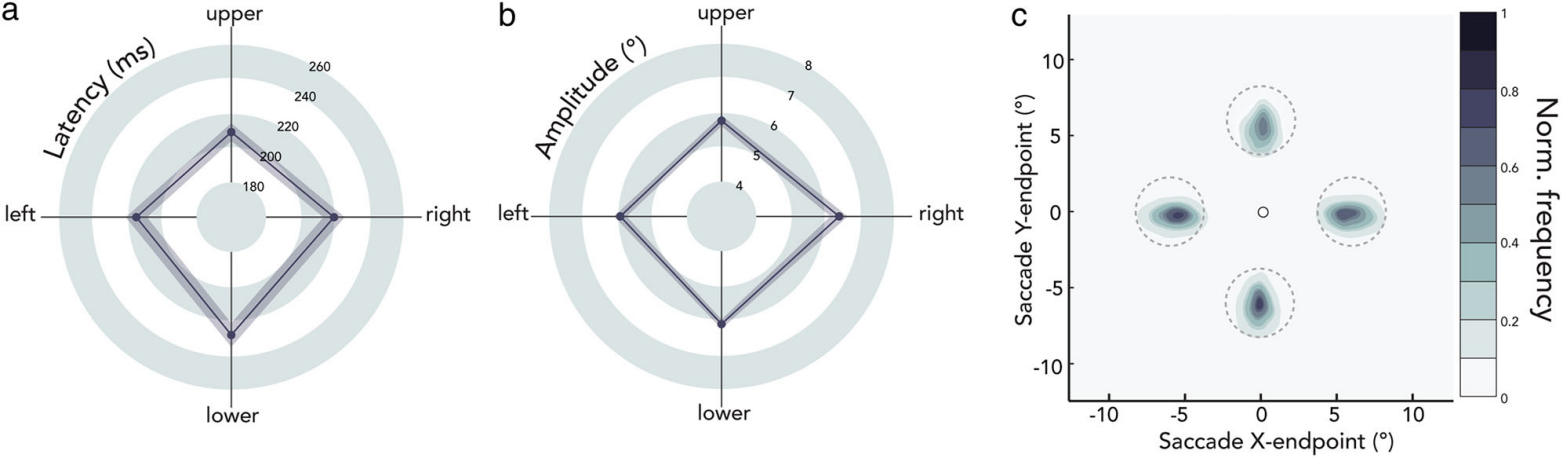
Group average saccade parameters as a function of saccade direction. ***a***, Saccade latencies (ms); ***b***, saccade amplitudes (°). Shaded error areas indicate ±1 standard error of the mean (SEM). ***c***, Normalized saccade endpoint frequency maps averaged across participants as a function of saccade direction. Dashed gray circles indicate the 2.25° radius saccade target region. The central black circle indicates the fixation location (not on scale).

We evaluated eye movement parameters as a function of saccade direction, to determine whether these parameters influence presaccadic attention effects at different locations (Fig. 8). A one-way repeated-measures ANOVA yielded a main effect of saccade direction on latency, i.e., saccade onset relative to cue onset (*F*_(1,11)_ = 22.459; *p *< 0.001; *η*^2^ = 0.672). Downward saccades were the slowest (mean = 228.015 ms; Fig. 8*a*), followed by horizontal (mean = 217.178 ms), then upward (mean = 209.159 ms) saccades (upward-downward: *t*_(11)_ = 5.902, *p *< 0.001, *d *= 0.909; horizontal-downward: *t*_(11)_ = 3.709, *p *= 0.002, *d *= 0.490; upward-horizontal *t*_(11)_ = 3.524, *p *= 0.006, *d *= 0.419). There was neither a main effect of saccade direction on saccade amplitude (Fig. 8*b*; *F*_(1,11)_ = 2.649; *p *= 0.088; *η*^2^ = 0.194; BF_10 _= 1.126) nor on precision—mean of the Euclidean distance between saccade endpoints and saccade target center (Fig. 8*c*; *F*_(1,11)_ = 1.327; *p *= 0.313; *η*^2^ = 0.108; BF_10 _= 0.458). Note that a minor difference in saccade latencies around the visual field has been reported previously (Honda and Findlay, 1992; Schlykowa et al., 1996; Tzelepi et al., 2010; Kwak et al., 2023). The differences in saccade parameters around polar angle did not parallel polar angle differences in presaccadic attentional benefits. Saccade parameters thus cannot account for the more pronounced benefits in contrast sensitivity at the horizontal meridian.

## Discussion

**Figure 9.**
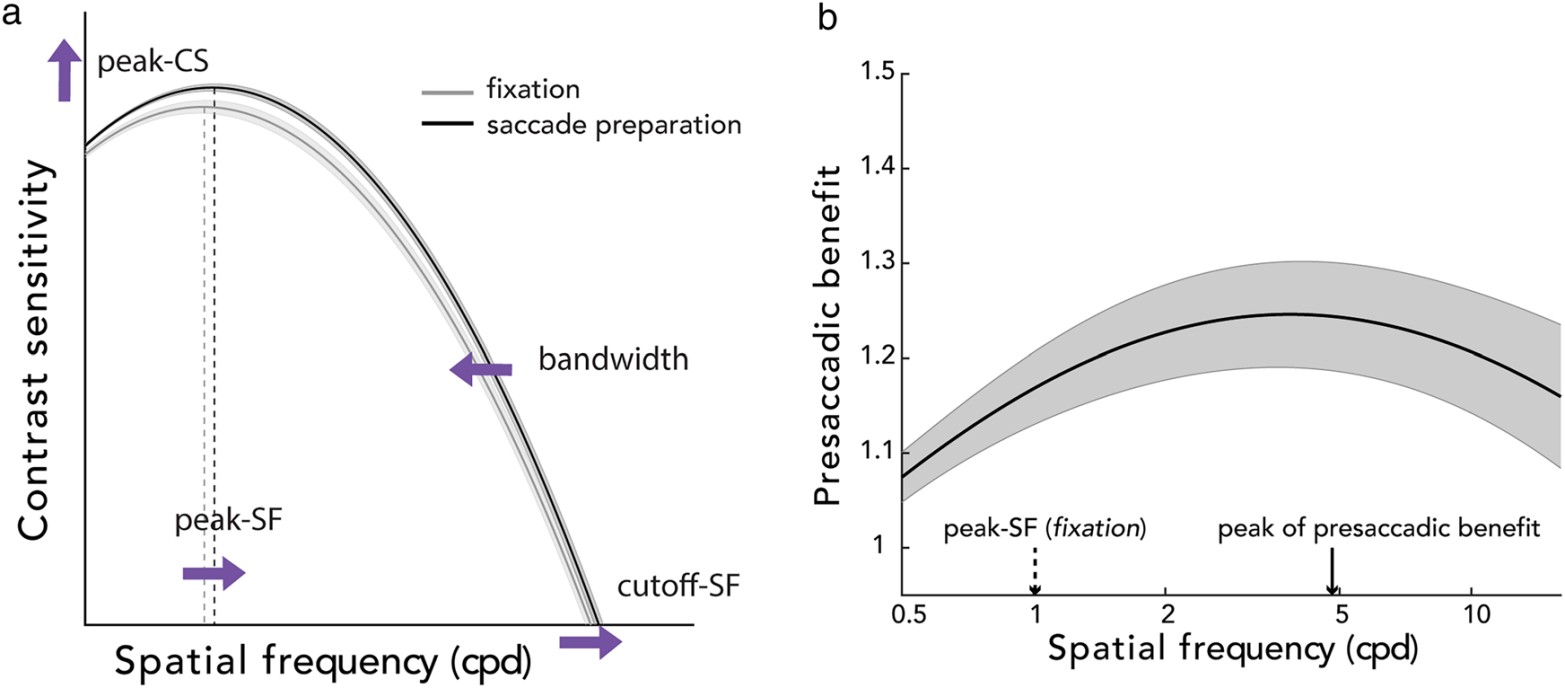
Summary schematic. ***a***, Key CSF attributes during fixation and saccade preparation. Presaccadic attention modulates peak contrast sensitivity (peak-CS), peak spatial frequency (peak-SF), cutoff spatial frequency (cutoff-SF), and bandwidth, which result in widening the window of visibility at the saccade target. Purple arrows demonstrate the direction of change. ***b***, Presaccadic benefit on contrast sensitivity across spatial frequencies. The benefit peaks at a spatial frequency (vertical solid arrow) higher than peak-SF during fixation (vertical dashed arrow). Shaded regions represent ±1 standard error of the mean (SEM).

This study reveals that presaccadic attention enhances and reshapes the CSF and that the magnitude of these effects varies around the visual field. We compared key attributes of the CSF, estimated through hierarchical Bayesian modeling (HBM), during fixation and saccade preparation for different polar angle locations. Presaccadic attention increased the peak-CS, shifted the peak-SF and cutoff-SF to higher spatial frequencies, and sharpened the bandwidth (Fig. 9), resulting in a larger area under the log CSF (AULCSF). Moreover, the increase in contrast sensitivity across a wide range of spatial frequencies, including the peak-CS, was more pronounced at the horizontal than the vertical meridian. Thus, during saccade preparation, the polar angle asymmetries in contrast sensitivity—specifically, the HVA (horizontal–vertical anisotropy)—are exacerbated.

These findings indicate that presaccadic attention renders the peripheral representation at the saccade target more fovea-like, in anticipation of the corresponding postsaccadic (foveal) input, which may facilitate a smooth transition in retinal images across saccadic eye movements (Herwig, 2015; Stewart et al., 2020; Li et al., 2021a). Increases in peak-CS, peak-SF, and cutoff-SF during saccade preparation resemble the way the CSF changes from peripheral to foveal locations. Peak-CS and peak-SF gradually increase from the periphery to the fovea (Hilz and Cavonius, 1974; Rovamo et al., 1978; Rovamo and Virsu, 1979; Jigo and Carrasco, 2020). Moreover, cutoff-SF (i.e., acuity) is directly proportional to the cortical magnification factor in V1 and increases from peripheral to foveal locations (Cowey and Rolls, 1974; Rovamo et al., 1978; Rovamo and Virsu, 1979; Virsu and Rovamo, 1979). The relation between bandwidth and eccentricity is not as clear in previous literature. Some studies demonstrate a sharpened bandwidth from the periphery to the fovea (Virsu and Rovamo, 1979; Goulet and Farivar, 2024), whereas others report no change in bandwidth across eccentricity (De Valois et al., 1982; Jigo et al., 2023). Therefore, whether the narrowing of the bandwidth with presaccadic attention is consistent with a CSF changing from the periphery to the fovea is yet to be determined.

Our findings are likely the perceptual consequences of neuronal modulations elicited during saccade preparation. First, relevant to the role of presaccadic attention in retinal stability, neurons predictively shift, or “remap,” their receptive fields (RFs) toward the location they will occupy postsaccade (Duhamel et al., 1992; Umeno and Goldberg, 1997; Neupane et al., 2016). Accordingly, peripheral stimuli at the saccade target, such as the test stimulus in our study, would have been processed by neurons more selective for foveal locations—with smaller RFs, higher SF tuning, and higher contrast sensitivity—during saccade preparation. Moreover, RFs shrink and shift toward the saccade target location, irrespective of the postsaccadic RF location (Tolias et al., 2001; Neupane et al., 2016). This leads to an increased proportion of neurons processing the saccade target. Both types of RF shifts predict the CSF parameter changes in the present study. Second, neuronal responses are enhanced, before saccade onset, when their RFs cover the saccade target location (Boch and Goldberg, 1989; Moore et al., 1998). This mechanism can be particularly related to the observed presaccadic enhancement of peak-CS, rather than of other CSF parameters in the SF space which likely rely on the abovementioned remapping mechanisms. Future studies should investigate whether distinct neuronal modulations underlie different perceptual changes during saccade preparation.

Our study reveals that the benefit of presaccadic attention on the CSF changes as a function of polar angle. During fixation, there were robust horizontal–vertical anisotropy (HVA) and vertical meridian asymmetry (VMA) effects in peak-CS, cutoff-SF, and AULCSF, but not in peak-SF, consistent with previous studies (Barbot et al., 2021; Jigo et al., 2023). During saccade preparation, the extent of the asymmetries for some of the CSF parameters was preserved; but for peak-CS as well as for a wide SF range, contrast sensitivity benefited more from saccade preparation at the horizontal than the vertical meridian, resulting in an even more pronounced HVA. Recent studies examining the interaction between presaccadic attention and polar angle asymmetries have yielded different results for contrast sensitivity (Hanning et al., 2022, 2024) and acuity (Kwak et al., 2023). The current study also uncovers a dissociation between location and instruction (fixation vs saccade preparation) interactions in peak-CS and cutoff-SF, which relate to contrast sensitivity and acuity measurements, respectively, within the same experimental design and participants: Asymmetries in peak-CS as well as contrast sensitivity at a wide SF range were exacerbated with presaccadic attention, expanding contrast sensitivity more across the horizontal than the vertical meridian, whereas those in cutoff-SF remained. Hence, the interaction between presaccadic benefits and polar angle asymmetries may depend on the visual dimension. Importantly, polar angle asymmetries in both dimensions (i.e., contrast, SF) are not alleviated even by the deployment of presaccadic attention: They are either intensified (Hanning et al., 2022, 2024) or preserved (Kwak et al., 2023). Similarly, polar angle asymmetries are alleviated with neither covert spatial attention (Cameron et al., 2002; Roberts et al., 2016; Purokayastha et al., 2021) nor temporal attention (Fernández et al., 2019).

What might cause the larger presaccadic benefit at the horizontal than at the vertical meridian? Our results suggest that horizontal and vertical saccades might recruit different neuronal populations during saccade preparation. In fact, there is evidence that a neuron's RF shifts toward the saccade target particularly for saccade directions aligned to the vector starting at fixation and ending at its RF center (Tolias et al., 2001). Therefore, it is possible that the presaccadic enhancement in the case of horizontal saccades arises from contribution of neurons with RFs along the horizontal, known to be more sensitive to various dimensions, than neurons along the vertical meridian, resulting in a larger benefit at the horizontal meridian. Although this account does not explain the dissociation between presaccadic enhancement in contrast sensitivity and acuity, our findings call for investigation of the neuronal populations active for different saccade directions and visual dimensions.

As both the CSF and the benefit of presaccadic attention vary as a function of polar angle, our study alongside previous reports (Hanning et al., 2022, 2024; Jigo et al., 2023) call into question the generalizability of behavioral and neurophysiological measurements obtained along the horizontal meridian, the most frequently assessed to characterize the effects of target eccentricity as well as saccadic eye movements. Future studies should test whether oculomotor feedback signals (between eye movement and visual areas), which are thought to underlie presaccadic modulations of visual perception (Moore and Armstrong, 2003; Ekstrom et al., 2008; Bisley and Mirpour, 2019; Hanning et al., 2023), differ between horizontal and vertical saccades, and whether other perceptual correlates of presaccadic attention—such as the sensitivity shift to higher SFs (Li et al., 2016, 2019; Kroell and Rolfs, 2021) or orientation tuning (Li et al., 2016; Ohl et al., 2017)—are more pronounced for horizontal than vertical saccades.

In this study, we have jointly examined key attributes of the CSF in a systematic manner, across the whole contrast range, a wide range of SFs, and critically at different polar angle locations. Our findings conceptually correspond to presaccadic attention effects on individual attributes of the CSF tested separately in various visual tasks and dimensions. Presaccadic attention enhances contrast sensitivity at the saccade target when measured at a fixed SF (Li et al., 2021b; Hanning et al., 2022). It also enhances sensitivity to SFs higher than the stimulus SF (Li et al., 2016, 2019), although this was tested only for a narrow frequency range (1–2 cpd) along the horizontal meridian. These results are in line with the rightward shift of peak-SF and cutoff-SF reported in the present study. Moreover, the increased cutoff-SF is consistent with presaccadic attention enhancing visual acuity (Kwak et al., 2023). Lastly, for a constant contrast level, saccade preparation benefits orientation discrimination at horizontal saccade targets primarily at mid-SFs (Kroell and Rolfs, 2021). Here, measuring the CSF, we show that presaccadic benefit on contrast sensitivity peaks at SFs higher than the peak-SF during fixation around the visual field.

Deploying covert attention without concurrent eye movements also modulates the CSF. With enough time to deploy endogenous attention, a voluntary and flexible mechanism, contrast sensitivity is enhanced across a broad range of SFs, at the peak-SF at each eccentricity as well as below and above it. Exogenous attention, an involuntary and inflexible mechanism, enhances contrast sensitivity primarily for SFs higher than the peak-SF at each eccentricity (Jigo and Carrasco, 2020). The presaccadic attention benefits in the current study are reminiscent of both types of covert attention. As for endogenous attention, contrast sensitivity benefits are present across a wide range of spatial frequencies, at the peak SF during fixation, as well as below and above it. As for exogenous attention, contrast sensitivity benefits are more pronounced for higher SF ranges during saccade preparation, with peak-SF and cutoff-SF shifting to higher frequencies. Compared with the changes brought about by these two types of covert attention, the change in CSF with presaccadic attention better mimics how the CSF changes from the periphery to the fovea: The CSF shifts rightward, but the benefit is not limited to a very narrow range at a spatial frequency higher than the baseline. Therefore, compared with previous findings on covert attention, the current results highlight the preparatory role of presaccadic attention for the future fovea.

In conclusion, our comparison of the CSF at fixation and during saccade preparation has revealed that presaccadic attention not only enhances but also reshapes our contrast sensitivity across different SFs at the saccade target. Counterintuitively, presaccadic attention enhances our window of visibility more effectively at the horizontal meridian locations, where vision is typically stronger. This finding suggests that the integration of pre- and postsaccadic visual representations by presaccadic attention results in a more efficient smoothing of vision with horizontal than vertical eye movements.

## Data Availability

The data is available on the Open Science Framework database (https://osf.io/m8ysa) upon publication.

## Synthesis

Reviewing Editor: Niko Busch, Westfalische Wilhelms-Universitat Munster Decisions are customarily a result of the Reviewing Editor and the peer reviewers coming together and discussing their recommendations until a consensus is reached. When revisions are invited, a fact-based synthesis statement explaining their decision and outlining what is needed to prepare a revision will be listed below. The following reviewer(s) agreed to reveal their identity: Anirvan Nandy. Note: If this manuscript was transferred from JNeurosci and a decision was made to accept the manuscript without peer review, a brief statement to this effect will instead be what is listed below.

#Synthesis

This manuscript has been transferred from the Journal of Neuroscience, where it has been reviewed by two experts on visual attention. The authors have revised the manuscript in response to the reviewers' comments.

One of the original reviewers assessed the revised manuscript again and recommended accepting it as is. I concur with the reviewers that the manuscript is well-written and methodologically sound. I am making the decision to accept this paper for publication based on the generally positive reviews and adequate responses and revisions provided by the authors to the reviewers' comments.
