# Supplementary figures and images for: Presaccadic Attention Enhances and Reshapes the Contrast Sensitivity Function Differentially around the Visual Field
Source: eNeuro. 2024 Sep 10;11(9):ENEURO.0243-24.2024. doi: 10.1523/ENEURO.0243-24.2024 (PMC11397507; doi:10.1523/ENEURO.0243-24.2024)

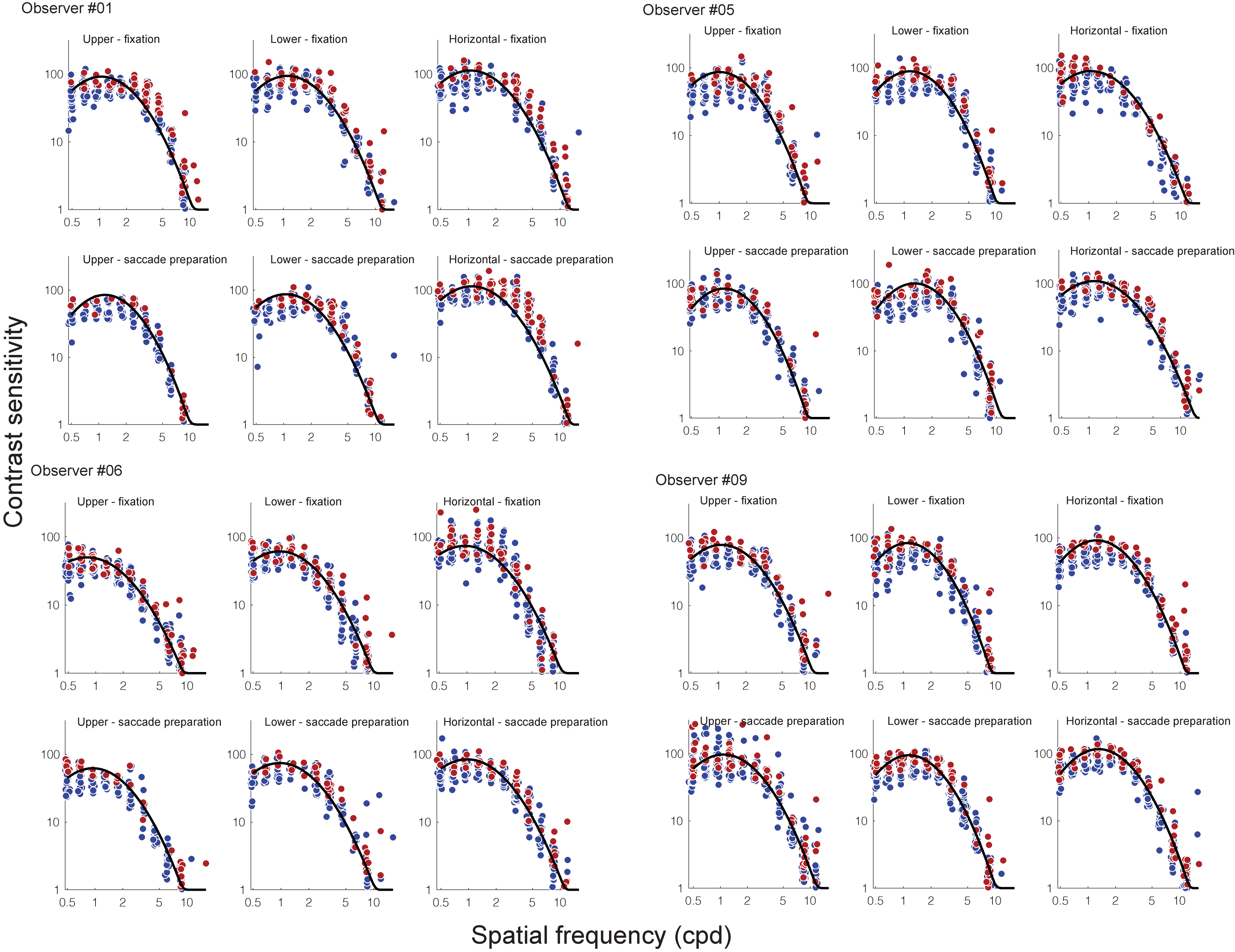

Supplement: Figure 2-1 — Individual observer data. The trial-by-trial datapoints and the model fits are shown for four observers, separately for each condition. Blue and red datapoints indicate trials with correct and incorrect responses, and the solid black line is the fitted HBM. Download Figure 2-1, TIF file. [file eneuro-11-ENEURO.0243-24.2024-s001.tif]

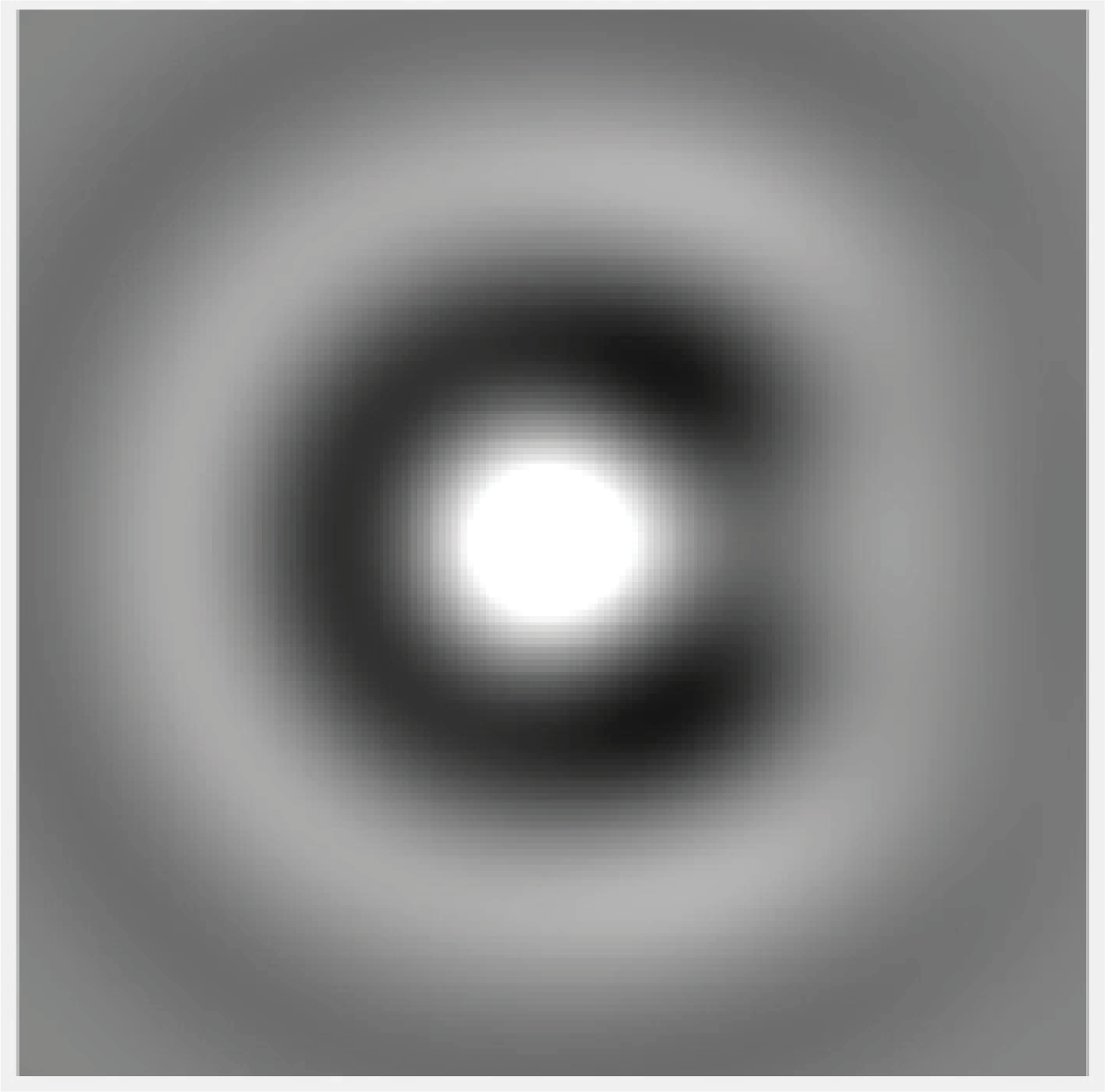

Supplement: Figure 4-1 — Demo of presaccadic effects on the CSF. A Landolt-C stimulus image was filtered with the group-average CSF parameters in the fixation and saccade preparation conditions. The demo changes from the perceived image during fixation to during saccade preparation, both at the horizontal location. (The demo was submitted separately as a .mov file) Download Figure 4-1, TIF file. [file eneuro-11-ENEURO.0243-24.2024-s002.tif]

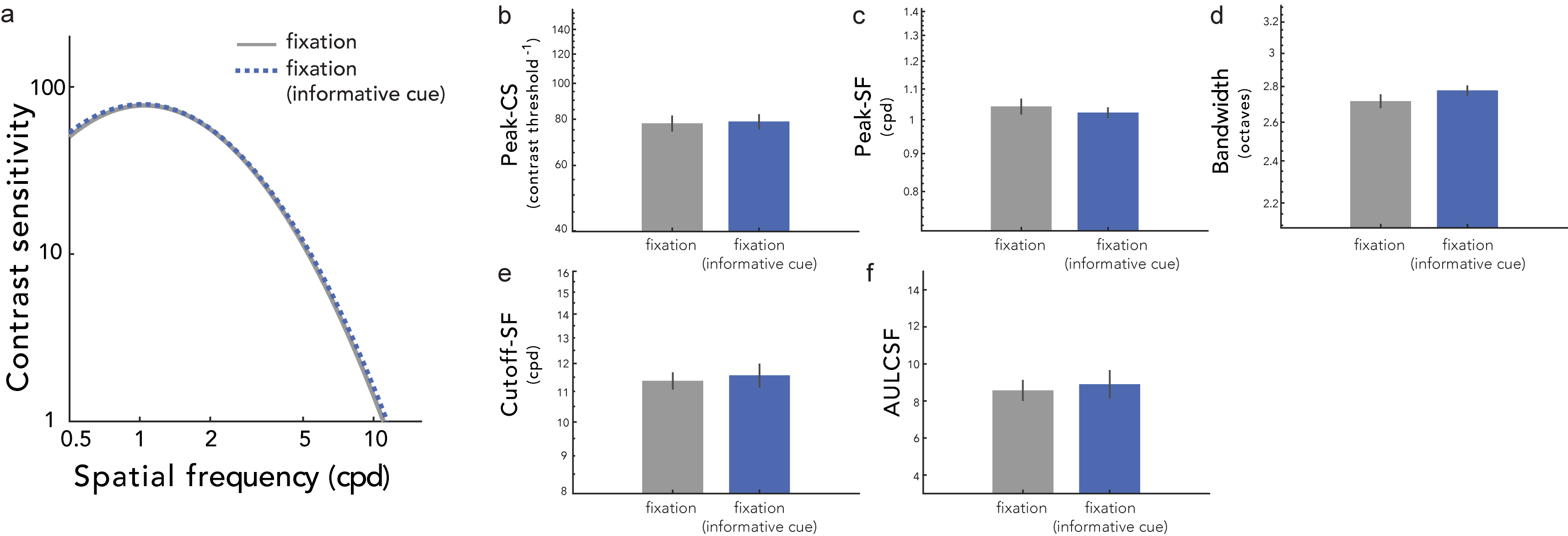

Supplement: Figure 5-1 — Results for the control condition. The control condition, in which participants (n = 5) maintained fixation while a spatially informative cue was presented, was compared with the fixation condition with a non-informative cue in the main study. The CSF and its key attributes did not differ between the two conditions. (a) Group average CSFs. (b-f) Group average key CSF attributes: (b) peak-CS; (c) peak-SF; (d) bandwidth; (e) cutoff-SF; (f) AULCSF. Error bars indicate ±1 standard error of the mean (SEM). Download Figure 5-1, TIF file. [file eneuro-11-ENEURO.0243-24.2024-s003.tif]
